# Memoires de l'Academie Royale de Medecine

**Published:** 1840

**Authors:** 


### Memoires de l'Academie Royale de Medecine.
### *Bailliere*, Paris, 1840.

These Memoirs may be considered as similar, in their object and general characters, to the Transactions of our Medico-Chirurgical Society.

To enable our readers to judge of the comparative merits of the two works, as well as to inform them of the progress of medicine and surgery among our neighbours, we purpose to give a condensed summary of the contents of the present volume.

These are as follow:—1. Historical *éloge* of M. *Itard*, the author of several well-known works on the Ear. 2. Historical *éloge* of M. *Laennec*. 3. Historical *éloge* of M. *Biett*. 4. Researches on the Diseases of Old Age, by M. *Prus*. 5. Treatise on the most common Diseases of Iceland, by Dr. *Thornstensen*. 6. Memoir on the operation of Lithotomy, by M. *Souberbielle*. 7. Memoir on the Dysentery of Guadaloupe, by Dr. *Cornuel*. 8. Researches on the structure of the Cortical Substance of the Convolutions of the Brain, by M. *Baillarger*. 9. Statistical Memoir on Pleuro-pneumonia, by M. *Pelletan*. 10. Memoirs, seven, on Poisoning—[*a*, poisoning from arsenic—*b*, on the means of ascertaining that the arsenic is not derived from the tests or vessels employed in the experiments—*c*, on a new process to discover the presence of arsenic absorbed into various organs of the body—*d*, on the arsenic naturally contained in the body—*e*, on the soils of cemeteries, and the arsenic which may be found in them—*f*, on poisoning from the tartrate of antimony—and, *g*, on poisoning from the salts of copper.] 11. Memoir on Re-vaccination. 12. New Researches on Human Urine, by M. *Lecanu*. And, 13, on Vaginal Cystocele operated on in a new method.

Passing over the three *éloges* with the single remark that, on the whole, we highly approve of such public panegyrics on distinguished characters, and that we should be glad to see the practice, carried although it be by our neighbours somewhat to excess, imitated in this country,* we shall at once proceed to the notice of the several practical memoirs whose titles we have just given, beginning with the—

### *Researches on the Diseases of Old Age.*

Dr. *Prus* has had unusually extensive opportunities of studying the diseases of old people, having for seven years past been attached to the medical staff of that immense establishment, the Hospice de la Vieillesse in Paris.

He first takes a rapid review of the various organic changes which gradually take place in the system as old age advances, and then points out the

---

* We observed by the public prints, that over the grave of our distinguished countryman, Sir Sidney Smith, who died within the last twelvemonth at Paris, three éloges commemorative of his private virtues and public services were pronounced, and that two out of the three were spoken by Frenchmen!—an act of generous and noble feeling. (*Rev.*)

practical conclusions which the enlightened physician will draw from the consideration of these in the treatment of its diseases. The imperfect mastication of the food in consequence of the loss of the teeth, the attenuation and atrophy of the muscular and mucous coats of the stomach and bowels, the diminution in the size and number of the lacteal vessels, and the contraction and induration of the mesenteric glands must necessarily retard and impede the formation and absorption of the chyle ; the progressive lesion of all the respiratory organs, the shrinking of the chest, the gradual wasting of the pulmonary parenchyma itself, and its diminished dilatability by the inspired air, the hardening and contraction of the larynx and bronchi, the thickening of their mucous covering, &c. all tend to render the aeration of the blood more and more imperfect; then the gradual changes in the substance of the heart and large bloodvessels,* too well known to all to require even a passing remark, the contraction and ultimate disappearance of the minute capillaries, to which is owing in a great measure the dryness and wrinkled state of the skin, the gradual enlargement of all the veins of the body, the inactivity of the lymphatic vessels in every part, the atrophy of the brain, spinal-marrow, and their nerves, the wasting of the muscles, the brittleness of the bones, not to mention numerous other organic alterations in every structure and tissue of the body—all these are most expressive indications that the machine of the body is becoming less and less able to perform its functions, and must therefore be less able to resist the encroachments of disease.

Besides these alterations in the solid parts of the body, equally obvious changes are going on in the composition and qualities of its fluids: these it is unnecessary to particularise; and we shall therefore now proceed to examine the interesting question, *What are the most common and most fatal diseases of old age?*

The following data will enable us to form an opinion on this subject.

Dr. *Prus* examined the bodies of 390 patients, between the ages of sixty and ninety years of age, who died in the *Bicetre* during the years 1832—3—4.

Of these, 149 died of diseases of the respiratory organs—viz. 77 of pneumonia, 26 of pleurisy, 18 of tubercular phthisis, 10 of asthma, eight of bronchitis, four of pulmonary congestion, two of asphyxia caused by excessive tympanitic distention of the abdomen, one of laryngitis, one of cancer of the larynx, and one of cartilaginous granulation of the lungs.

---

* Dr. *Prus* remarks that, in consequence of the various changes which age induces in the arteries, we should always examine the state of the pulse at the heart, and not at the wrist, in old persons. " How often," says he, " have patients, whose radial pulse was feeble and irregular, but whose heart announced an energetic resistance, been bled with marked advantage, and thus escaped a speedy and inevitable death." (This precept requires to be received with caution; a strong tumultuous action of the heart is not unfrequent in old people, especially when there is incipient hypertrophy of its ventricles or contraction of its orifices, (a common occurrence,) which by no means indicates the necessity for depletion; and even when this is deemed necessary, it is often safer to draw blood by cupping over the cardiac region than by opening a vein in the arm.—*Rev.*)

Lesions of the nervous centres are next in point of frequency; 101 deaths being attributable to this class—viz. 25 to meningitis, 23 to cerebral ramollissement, 18 to recent cerebral apoplexy, six to apoplexy of old date, six to meningeal apoplexy, five to cerebritis, (in which purulent matter was found blended with the substance of the brain,) four to serous apoplexy, four to *coups de sang* or sanguineous congestions without laceration of the cerebral substance, two to capillary apoplexy of the convolutions, two to apoplexy of the annular protuberance, one to apoplexy of the cerebellum, one to contusion of the brain, and one to general paralysis.

Then follows the class of diseases of the circulatory organs, which amounted to 64. These cases may be subdivided as follows:—54 of diseases of the heart, three of arteritis, or arterial ossification giving rise to dry gangrene of the extremities, two of aneurism of the aorta, two of pericarditis, one of obstructed vena cava from an enlarged lumbar gland, and one of vegetations in the aorta, accompanied with periostosis of the clavicles.

The diseases of the alimentary canal amounted to 49;* there being 27 cases of enteritis, 10 of cancer of the stomach, four of gastro-enteritis, three of colitis or dysentery, two of gastritis, two of diarrhœa without inflammation, and one of softening of the mucous membrane of the stomach.

In addition to the causes of death enumerated, eight patients died of diseases of the liver, and the remaining 19 either of erysipelas, nephritis, fever, or some other casual malady.

We need scarcely mention that, in almost every case, the disease which appeared to be the more immediate and primary cause of death was seldom solitary, but was accompanied with morbid changes in other parts of the body at the same time.

The frequency of certain diseases, especially of inflammatory diseases of the lungs and also of apoplexy, will be found to vary very considerably in different years: hence the importance of attending to what has been called the *medical constitution* of the seasons on the development of certain maladies. Dr. *Prus* insists particularly, and with much propriety, on the necessity of aged people being warmly clad, and of guarding as much as possible against the influence of atmospheric vicissitudes. Neglect of these precautions is, he thinks, one of the chief causes of the great frequency of pneumonia among the inmates of the Bicetre and the Salpetriere hospitals at Paris.

So much for the relative frequency of the diseases in the different systems of the body in old age: let us now endeavour to determine the average number of deaths or the ratio of mortality among sick persons above sixty years of age.

Dr. *Prus* states that in the course of three years he lost 430 patients out of 1345 received upon the sick list at these two immense establishments. The mortality in the practice of the other physician, his colleague, was quite as great; so that we have a total of about 860 deaths in the course of three years, or of 287 yearly, among 2500 persons—the usual number of the

---

* It was long erroneously imagined that the most frequent diseases of old age were those of the abdominal viscera.

resident inmates ; giving the average or ratio of about one to eight and four-fifths, for the entire number of these inmates; and of one to three and four-eighths for the 1345 patients who were under medical treatment.

Dr. *Prus* proceeds next to give several tables to illustrate the actual and relative mortality in the different months of the year : suffice it to say that the greater number of deaths occurs during the cold seasons. He then enumerates the diseases of 685 patients who were cured or relieved during his three years' practice at the Bicetre and Salpetriere hospices. Of these there were 216 cases of affections of the respiratory organs, 151 of affections of the nervous centres, 144 of abdominal complaints, 54 of diseases in the organs of circulation, 22 of cutaneous affections, and the remaining 98 of diverse and occasional diseases.

Having treated of these subjects, our author makes some useful remarks on the great difficulty of accurate diagnosis in many of the diseases of old age.

"What must frequently astonish the physician," says he, "is the want of re-action in most organs, and even in those which are immediately connected with life. For example, the lung may pass into a state of grey induration, and the stomach may be the seat of a cancer, without the attendance of any of those symptoms which almost invariably accompany them at earlier periods of life. The heart itself, as long ago remarked by *Bichat*, is often found in old age to exhibit lesions which would have quickly killed an adult or child, although during life no disease may have been suspected. Hence the necessity of a most watchful care in the examination of old people's maladies......... How much has auscultation done in reference to the lesions of the respiratory organs! Before the knowledge of this great discovery, the diagnosis of many thoracic diseases among the aged was almost quite impossible ; a circumstance which explains how such a man as the illustrious *Pinel*, who had passed many years of his life in the Hospices de la Vieillesse, has described under the name of adynamic fever a state which is now recognised by all pathologists as belonging to the second and third degrees of senile pneumonia."

Another circumstance with respect to the diseases of old age, which is no less remarkable, is that the mutual sympathy and co-operation of the different organs for the preservation of life are greatly impaired. Each organ seems to live isolated; and hence it may succumb to disease, without the other organs coming as it were to its assistance. Thus the lung may become impermeable to air and even completely disorganised, and yet the heart will often not announce the existence of any lesion by an increased frequency or by any other change of the pulse.

"I have often," says Dr. *Prus*, "made a comparison which seems to me exact. Go into a ward devoted to the treatment of the diseases of old age, and you will be astonished at the complete indifference with which a patient witnesses his neighbours and co-inmates die around him. It is the same in the economy of the old man ; it will become demolished piece by piece, without there being any re-action of the *ensemble*, or without any preservative effort being made by the system."

In a future paper Dr. *Prus* proposes to describe the leading specialties or peculiarities which he has observed in some of the most frequent and most fatal of the diseases of old age.

*Memoir on the Operation for Stone.*

M. *Souberbielle* is one of the most, if not the very most, experienced and successful lithotomists of the present day in France. He has during his practice performed the operation several hundreds of times, and within the last five years has operated on fifty patients. The present memoir is occupied with the histories of these fifty cases, and with practical remarks on their general results. These we shall now briefly present to our readers; and it is the more important that the opinions of M. *Souberbielle* be generally known, as he differs from the leading surgeons of other countries as well as of France in giving a decided preference to the *high* over the *lateral* operation of lithotomy.

With respect to the *age* of the patients, nine were under ten years, three above ten and under twenty-two years, one was forty-two years old, five were from fifty to sixty years, thirteen were sexagenarians, seventeen were septuagenarians, and two were octogenarians.

M. *Civiale* is probably mistaken when he asserts that one half of the entire number of calculous patients are children: the disease seems to be most frequent at the two extremes of life.

The mortality in these fifty cases amounted to eleven—which is rather greater than usually attends the practice of M. *Souberbielle.* This was chiefly owing, he thinks, to the circumstance of the operation of lithotrity having been of late years more frequent than it used to be; and as this operation is performed only in favorable cases, the chances of success for the cutting operation in the remaining cases are proportionally less. M. S. adds that if he merely consulted his reputation as a successful lithotomist, he should certainly not have performed the operation in at least three of the fifty cases, as the health of the patients was at the time very bad, and there were grounds to suspect organic changes in the urinary organs.

The average success of his practice has been very nearly the same as that which attended *Frere Come;* this celebrated surgeon lost nineteen out of a hundred cases after the high operation of lithotomy. Indeed this seems to be about the average mortality after the operation of lithotomy, whether by the high or by the lateral method, in the practice of the most distinguished lithotomists, whenever a large number of cases is compared.* The wonderful success attributed to *Raw,* who it has been said cut 1550 patients without losing one, has been shewn by *Sandifort* to be quite fabulous; as it seems that he performed lithotomy no fewer than 2200 times in all. M. *Souberbielle* himself has operated successfully in 29 cases following; but then at other times he has been greatly less fortunate; so that, as he frankly admits, the average mortality in his practice has been one death in every five or six cases—a ratio which he believes, as we have already stated, to be about the average one after the operation of lithotomy, whenever a large number of cases is compared.†

* *Dupuytren* has expressed the same opinion in his Treatise, which was published a few years ago.

† It seems that the Paris hospital surgeons are unusually unsuccessful in their operations of lithotomy. If we are to believe M. *Civiale,* of 61 cases at La Charité, 35 proved fatal; of 96 at the Hôtel Dieu, 27 were fatal; of 11 at

As to the *sex* of the patients in M. *Souberbielle's* 50 cases, 48 occurred in men, and two in women. During his long and very extensive practice he has performed lithotomy fifteen times on females—nine times by the high, and six times by the low operation.* Two cases proved fatal; once after the high operation, in consequence of suppurative inflammation in the kidneys; and in the other case, after the low operation, in consequence rather of excessive exhaustion and debility than of the operation itself. The size of the calculi was in most of the cases (in females) large; one weighed five ounces and a half; two weighed four ounces; one three ounces and a half, and two three ounces. In two cases only was there more than one calculus present; whereas in 16 of the 48 cases in the present series, which occurred in males, there were several calculi in the bladder.

In all the cases of the high operation on women, M. Souberbielle has used the *sonde à dard* introduced along the urethra; and on every occasion, whether of the high or of the low operation, a flexible catheter was left in the bladder for several days after. The low operation—which is applicable whenever the calculus is small, or when it is at all engaged in the vesical orifice of the urethra—is best performed with the *lithotome caché;* the cutting edge being turned to the left side, and the instrument then withdrawn transversely so as to divide the left side of the urethra and of the neck of the bladder.

So much for the operation in women. Let us now continue our general remarks on the histories of the 50 cases which form the subject of M. *Souberbielle's* memoir. In 12 of the cases, unsuccessful attempts to remove the calculi by lithotrity had been previously made; and in the majority of the 38 remaining cases this operation had been deemed inapplicable.

In 40 out of the 50 cases, the high operation was performed; in the remaining 10, and of these eight were in children, the calculi were extracted by the lateral operation. In the case of the two adults, M. *Souberbielle* preferred the lateral operation in consequence of there being a partial paralysis of the bladder (which ceased on recovery from the operation) in one of them, and in the other, which occurred in a woman, because the calculus was already impacted in the urethra.

Except in the case of children, or when there are some peculiar circumstances, M. *Souberbielle* gives a decided preference to the high over the low or lateral operation of lithotomy.

"This preference," says he, "is founded alike upon reasoning and upon the results of experience. By the high operation we can easily extract every sort of calculi, whatever be their size, their number, their situation, the volume of the prostate gland, &c. &c. Besides, the operation is less painful, and is not apt to be followed by the same accidents, since only the integuments, the linea alba, the adipose cellular tissue, and the body of the bladder are divided; and there

---

Beaujon, 6 were fatal. M. *Souberbielle* adds—" I am far from guaranteeing the accuracy of these assertions; but I have certainly been much struck with the sad want of success after lithotomy in the hospitals of Paris."

* As a general rule, M. *Souberbielle* prefers the high operation in women as well as in men; in the former, because, says he, " it is not apt to be followed by incontinence of urine, so common after the low operation, especially when the calculus is large."

is not the same danger of hæmorrhage, fæcal fistulæ, infiltrations, seminal impotence, &c. taking place.

The operation in itself is very seldom the cause of death, as is distinctly proved by the results of the post-mortem examinations in fatal cases. The cause of death is usually attributable to the exacerbation of pre-existent organic disease, either in the bladder itself or in the kidneys."

With respect to the *size* and the *number* of the calculi in M. *Souberbielle's* 50 cases, we may mention that in three of them the calculi were very large; in one, which occurred in a medical man 82 years of age, the calculus weighed half-a-pound; and in another case, which however unfortunately proved fatal, upwards of 300 calculi were extracted. Our author is of opinion that when calculi are either encysted or firmly grasped and retained by the coats of the bladder, they are much more easily extracted by the high than by the lateral method of operating, and appeals to six cases in his present catalogue in proof of this assertion. "In one case especially the calculus, being lodged in a cavity at the fundus of the bladder, could not have been reached from the perineum, and the manœuvre by which it was turned out from its place must have been quite impracticable."

This circumstance of the *fixité* of the calculi in the bladder, which in M. *Souberbielle's* opinion gives so many advantages to the high over other methods of operating for stone, is far from being so unfrequent as is often supposed; for in 16 out of the 50 cases, the calculi were either firmly embraced by the parietes of the bladder, or lodged in cavities, the edges or circumference of which in two cases required to be divided with the bistoury. In one case, where the pelvis was so much deformed that the lateral operation could not have been performed, three calculi, each of the size of a chesnut, were successfully extracted.

As the observations of so experienced a man as M. *Souberbielle* must be interesting to every practical surgeon, we shall follow him in his comments on the objections which are usually made to the high operation: these are, the risk of the infiltration of urine, of wounding of the peritoneum, and that of hæmorrhage.

As to the first of these accidents, which is certainly one of the most frequent and dangerous consequences of the lateral operation, it occurred in *one only* of the forty cases where the high operation was performed; and "I may state," continues our author, "that in my very numerous operations I have never known it to happen except in this single case, where it was attributable to a circumstance foreign to the operation." He asserts that this accident can scarcely occur if care be taken not to separate the anterior wall of the bladder from its adhesions to the neighbouring parts; and he therefore very strenuously condemns an advice given by Ludwigg and some other surgeons to tear, and not to cut, the cellular substance behind the linea alba.

With respect to the risk of wounding the peritoneum in the high operation of lithotomy, we find the following remarks:—

"However little enbonpoint there may be, the place of the reflection of this membrane is from two to two and a half inches distant from the superior angle of the incision, and it is very rare that this interval is less than one inch: moreover, in dividing the linea alba with caution, and as Frere Come says, rather by a pressing than by a sawing movement, the aponeurotic band being strongly stretched, the peritoneum must retreat from the cutting edge of the instrument, and a wound of it supposes some rare condition of the parts, such as excessive emaciation, or an unusual contraction of the size of the bladder. (This sentence is far from being clear.—*Rev.*). But even when the peritoneum has been wounded, there is by no means that amount of danger which is usually imagined, provided the discharge of the urine is maintained, by means of a syphon introduced into the bladder, for some time after the operation. The accident occurred in two of the forty cases; and although one of these two cases proved fatal, the death was certainly not attributable to any effusion of urine into the peritoneal cavity, but to the co-existence of serious disorganization of the kidneys and bladder. In the other case, no unpleasant consequences followed."

The risk of fatal or even troublesome hæmorrhage after the high operation is exceedingly small. Nevertheless one of the forty cases proved unfortunate from this rare accident; the following are the particulars.

*Case.*—A gentleman, 58 years of age, excessively fat and subject to hæmaturia, had undergone several unsuccessful attempts of lithotrity, when he applied to M. *Souberbielle* to relieve him of a stone in his bladder. The high operation was performed, and two rough calculi were extracted. After the operation the urine was very bloody, and the patient was much distressed with frequent calls to pass it. A large quantity of clotted blood came away with injections, and then fluid blood flowed out in a continuous stream from a catheter left in the urethra. The extreme corpulency of the patient prevented an accurate examination of the parts to ascertain whether the bladder was distended or not. The patient becoming exceedingly faint and low, M. *Marjolin* was called in to the consultation ; but nothing that was tried was of any avail in restraining the hæmorrhage, and the patient died twenty-four hours after the operation. On dissection the bladder was found to be greatly dilated, and to contain not less than two pounds of coagulated blood ; the cellular substance also behind the abdominal muscles, even as far as the umbilicus and the iliac fossa, was infiltrated with blood. The hæmorrhage seemed to have come not from any considerable vessel, but from a vascular state of the bladder itself, which was throughout much thickened and hypertrophied.

We have already stated that M. *Souberbielle* always makes use of the *sonde à dard* in performing the high operation, whenever he can. He employed it in 37 of the 40 cases. In the three other cases, the contracted state of the bladder and its close application round the calculus prevented the introduction of this instrument, and the operator was therefore obliged to cut directly upon the stone itself, and then enlarge the incision either upwards or downwards with the *bistouri caché* or with the *bistouri lenticulé*.

After the operation M. *Souberbielle* always leaves an elastic gum syphon in the bladder, to evacuate the urine as it is secreted: he has never met with any inconvenience, but on the contrary has always witnessed great advantage, from this practice. When the urine escapes by the wound, it is usually about the eighth day, it then continues to do so for about a week, and generally ceases about the fifteenth day ; often however, none passes at all this way; and in one of the cases the wound healed by the first intention, and the patient voided his urine by the urethra on the third day. It is worthy of remark that the patients seldom or indeed never experience so much pain when the urine escapes by the wound after the high operation as after the lateral one, especially when the edges of the wound in the perineum have been bruised by the instruments, or by the calculus itself during the process of extraction.

The syphon was usually kept in the bladder from fifteen to twenty or twenty-five days. Before removing it, M. *Souberbielle* recommends, that for several days previously, it be plugged during three or four hours at a time, for the purpose of ascertaining the solidity of the cicatrix of the bladder: whenever it incommodes the patient, or the contractions of the bladder force it out, it may be withdrawn. The only inconvenience of this, which M. S. has observed, is that the healing of the wound is somewhat retarded.

In concluding his memoir, our author mentions that seven out of the fifty patients, on whom he operated, were affected at the same time with hernia—a proportion which is higher than the average frequency of rupture in other individuals. It is probably from the violent contractions of the bladder and of the abdominal muscles to expel the urine in calculous diseases, that such patients are apt to become ruptured. Now the effect of such a complication must be to draw down the peritoneum lower than it is in a healthy state of the parts; and hence, although its existence does not constitute a sufficient objection by itself to the performance of the high operation, yet more than usual caution should be used by the surgeon in cutting into the bladder in such cases.

In ten of the forty cases the patients were very fat; this circumstance does not increase the difficulty of the high operation of lithotomy nearly so much as is supposed, although certainly the extraction of the stone is somewhat *gené* by a fatty condition of the parts; but then, on the other hand, in such cases there is less risk of wounding the peritoneum, and moreover the cellular tissue, when filled with adipose substance, can be divided with greater ease and neatness, and is much less likely to admit the infiltration of urine into it than when it is more lax.

The occurrence of vomiting during the first few days after the operation—and indeed the same is apt to occur after the lateral operation—by no means implies the accession of peritonitis: this symptom often subsides spontaneously or under the use of opiates.

Lastly, it is worthy of notice in reference to M. *Souberbielle's* fifty patients, that the operation was performed on two who had undergone it some years previously. He remarks—"I have in general been successful with my second operations. The most remarkable cases on this head, which have occurred in my practice, are those of MM. Suire, Daumy and Seraphin, each of whom I cut for the stone four different times."

Such is a brief summary of the contents of M. *Souberbielle's* memoir, which well deserves to be consulted in the original by all operating surgeons. The practice in some of the cases seems to have been rash, as the prognosis was far too unfavorable to justify the performance of so serious an operation as that of lithotomy, either by the high or by the lateral method. The success however on the whole, even under such disadvantageous circumstances, sufficiently shews that the high operation in the hands of a dexterous surgeon is by no means so dangerous as it is generally believed to be. We again recommend the attentive perusal of the memoir to all lithotomists.

The next memoir which comes under our notice is one *On the most frequent Diseases in Iceland*, from the pen of a Dr. *Thornstensen*, a Danish physician, who has for many years been resident there: it is written in Latin. As might be supposed, inflammatory, febrile, and rheumatic complaints form the staple of the nosological catalogue among the hardy Icelanders: syphilis, it is stated, is unknown; scurvy is not unfrequent.

The following extracts on a disease, which is common in hot climates but rare amongst us, are interesting:—

"The most frequent of all spasmodic diseases in Iceland is the Tetanus of new-born infants, which is almost endemic in the adjoining island of Vestmanney. It usually commences on the fifth, sixth, or seventh day after birth, with a rigidity of the muscles of the nucha, at first temporary and occasional, and then more continued; the rigidity extends to the jaws and throat, so that deglutition becomes difficult, and the mouth can scarcely, or not at all, be opened. Gradually the muscles of the back become tetanically contracted; then convulsive motions arise, during which the head is powerfully drawn backwards, and the face becomes livid and convulsed. These attacks return more and more frequently, and are now of longer duration, and at length every part of the body becomes stiffened with spasm. In almost every case the infant dies before the close of the seventh day after birth.

Nearly three-fourths of all the infants—usually about twenty per annum—born in the island of Vestmanney, perish from this disease, and in Iceland not more than one in ten infants, who are affected, is saved.

It is difficult to account for the much greater frequency of the disease among the inhabitants of the one island than of the other. Some have conjectured that it is owing to the people in Vestmanney living more upon certain sea-birds.

The treatment of this disease is exceedingly difficult. Internal remedies seem to have little effect: what has proved most useful in my practice is the rubbing in of an ointment, consisting of one ounce of the ung. hydrarg. cinerei and two drachms of powdered opium, after the administration of purgative enemata, upon the neck and back every hour: the internal exhibition of the tincture of musk at the same time may be resorted to with advantage."

Among the more frequent *chronic* diseases among the Icelanders is *induration of the liver:* we certainly were not prepared to expect that a disease, which is usually supposed to appertain more particularly to hot climates, was of so frequent occurrence in the frozen regions of the north. The following description of it by our author is worthy of notice.

" Induration of the liver is a very common (admodum vulgaris) disease in this island. We meet with numerous invalids who, for a number of years, sometimes from an early period of life, have suffered from a dull oppressive pain in the right hypochondrium, which is usually found to be more or less distinctly enlarged. They are usually affected with dyspepsia, constipation, loss of appetite, and general debility. In many cases, the swelling of the side, after extending more and more until almost the entire abdomen becomes occupied, becomes soft and fluctuating. The induration has then been followed by suppuration; and the contents of the hepatic abscess may at length be discharged either into the cavity of the peritoneum, or outwardly at the umbilicus or at some other point of the abdomen. I have often opened these abscesses with the knife, and given exit to an almost incredible quantity of most fœtid pus, blended in some cases with hydatids and loose floating substances, like partly-dissolved cysts: these cases have usually done well ultimately. In other instances the hepatic induration never passed into the suppurative process, but the greater part of the viscus is converted into a large steatomatous mass. On examining the abdomen after death in such cases, we often meet with numerous hydatidiform bodies, either attached to the liver or floating loose. In a very remarkable case of hydatidic degeneration of the hepatic parenchyma, which occurred in a man 30 years of age, the liver was found on dissection to be immensely enlarged and looked like a huge leather bag filled with water; on dividing it, nearly twenty pounds of pure serum flowed out.

These diseased states of the liver often commence in youth, and continue for a great number of years before the viscus becomes seriously altered."

Another Icelandic disease is one, too, whose *habitat* we usually associate with warm climates—*Lepra orientalis.*

"I have observed," says Dr. *Thornstensen,* "three species of the disease here. The *first* and most frequent of these is characterised by the following symptoms: impairment of all the mental and bodily faculties, roughness of the voice, a scorbutic livid aspect of the countenance, fætor of the breath, and a greater or less loss of the sensibility of the surface, and of the sense of touch. In various parts dark-coloured patches make their appearance on the skin, which is there always somewhat raised and more or less insensible; these spots or livid tubercles are sometimes observed on almost every part of the body, face, and extremities.

After a time, they pass into fætid lardaceous sores, which in some cases are attended with pain, and in others with a want of feeling in the parts. The whole organism of the body becomes vitiated, the senses of sight and hearing become weaker and weaker, the eyes are sometimes utterly destroyed, and the cavities of the orbits are occupied with foul ulcers, the bones of the nose and palate become soft and carious, and occasionally the brain itself is involved in the general morbid decay. The duration of this form of lepra varies from two to ten years or upwards: from the character of its symptoms I have been in the habit of designating it the Scorbutic Lepra.

The *second* species is called by the natives *limafallssyki,* Lepra decidua; its diagnostic character being the tendency of the fingers and toes to mortify and fall off. The disease commences with loss of sensibility in the fingers or toes of one of the hands or feet: there is usually no swelling, but there is always a burning pain around the affected part; the integuments burst, an ulcer is formed, and, after the lapse of some time, one or more bones come away in a carious state.

The surface of the body, at the same time, is usually the seat of a squamous eruption, and the muscles of the extremities become excessively wasted and feeble; the functions, however, of the brain and other viscera &c. are not apt to suffer. So great is the insensibility of the limbs in some cases of this form of lepra, that the patients feel little pain from amputation of the diseased extremity; the substance of the muscles is found to be converted into a semi-cartilaginous tissue, and the divided arteries pour out very little or no blood. Notwithstanding these serious changes, the stump will often heal favourably; and, even after the loss of more than one limb, patients have been known to live for several years without any severe suffering.

The *third* and rarest form of lepra met with in Iceland is most closely allied, if not identical with the *Lepra Arabica.* The integuments of almost every part of the body become indurated, squamous, insensible and elephantine; the muscles and all the other organs are attenuated and wasted, although the appetite usually remains good; and the cerebral functions are always more or less impaired. There is no tendency to the formation of ulcers, as in the preceding form of the disease."........

The following memoir is one by Dr. *Cornuel on Dysentery*, as observed by him at Guadaloupe.

There is little that is novel or very interesting in it, and we shall therefore only allude to one or two of the author's remarks on the treatment of some of the forms of the disease.

Opium is certainly the most important of all remedies, and is required in almost every variety of the disease. As a matter of course, its administration as to dose, frequency of repetition, and so forth, must depend on the severity and peculiarities of each case. The combination of ipecacuan and opium is of especial benefit in numerous examples. Of late years, a pill containing these two ingredients with calomel, and known by the name of the *English pill*, has been used with remarkable success at Cayenne by Dr. *Segond*, and more recently by Dr. *Cornuel* at Guadaloupe. The formula is eight grains of powdered ipecacuan, four of calomel, one of the watery extract of opium,—to be divided into six pills; one to be taken every two hours.

In some of the milder and more chronic forms of dysentery, Dr. *Cornuel* has found much benefit from the use of mild aperients, such as Epsom salts in the dose of one or two drachms dissolved in four ounces of herb-tea, or manna in the dose of half an ounce, taken early in the morning; an opiate having been administered on the preceding evening at bed-time. The chloruret of sodium, with small doses of morphia, was administered both by the mouth and in enemata with excellent effects, when there was reason to believe the presence of slight ulceration of the intestinal mucous membrane.

*Researches on the Structure of the Cortical Substance of the Cerebral Convolutions*, by Dr. *Baillarger*.—The object of the author is to shew that the cortical surface of the nervous centres is distinctly stratified, consisting of several alternating layers of grey and white medullary matter, and that these strata may be aptly compared to the alternating plates of different metals or other substances in a Voltaic pile.

After minutely describing numerous anatomical examinations of the cortical substance of the brain in man and in the lower animals, he sums up the results of his enquiries in the following, among other, conclusions:—

"1. The cortical substance of the cerebral convolutions consists of six layers alternately grey and white, proceeding from within outwards. If we examine a thin slice of the grey substance placed between two pieces of glass, the six layers appear alternately transparent and opaque.

3. The white strata, which exist in the thickness of the cortical grey substance, are formed of two rows of vertical fibres.

14. The superposition of six strata, alternately of white and grey nervous matter, in the cortical substance of the brain, suggests the idea of a galvanic pile.

15. This analogy between the structure of the cerebral surface and the arrangement of a galvanic apparatus may be adduced as an argument in favour of the following two propositions.

The nervous, like the electrical action, is in relation to surfaces and not to masses. The nervous influx, like the electrical stream, is transmitted by surfaces."

The opinion that there is some analogy between electricity and the mysterious cause of nervous action is now admitted by many of the leading physiologists of the present day. It is therefore reasonable to expect that there are some features of resemblance between the anatomical arrangements of the nervous centres and those of a galvanic apparatus. Different opinions have been held by different anatomists as to the part of the nervous system which may be most aptly compared to a pile.

*Rolando* fixed upon the cerebellum ; he says—

"If an apparatus composed of different non-metallic substances, such as schistus, charcoal, muscular substance, and cerebral substance, and if the electrical organ of the gymnotus and torpedo, composed of an albumino-cartilaginous and other similar substances, is known to generate a great quantity of electrical fluid, why may not a similar principle be developed by the numerous layers of the yellow and cineritious matter of the cerebellum?"

Our author, while he does not impugn the accuracy of *Rolando's* conclusion with respect to the cerebellum, alleges, with considerable shew of reason, that, if electrical action is developed by the nervous centres, it is much more probable that the seat of this development is not in one part only, but rather that it is diffused over their entire extent. "Assuredly," says he, "if the Italian anatomist has been led from the circumstance of the superposition of two laminæ of nervous matter to affirm that the cerebellum may be compared to a voltaic pile, how much more readily must he have admitted the same thing for the brain, had he been aware of the stratified structure of its surface, in which I have shewn that six alternating laminæ can be proved to exist?" He adds, "in a future memoir I will shew that a similar arrangement can be demonstrated in the surface of the cerebellum itself, and also of the spinal cord; so that this stratified structure is common to the entire extent of surface of the nervous centres. But it is not on the surface alone of these centres that such an arrangement exists ; it is well known to be quite obvious in the substance of the corpora striata, the tubercula quadrigemina, and in the tuber annulare also."

In confirmation of his views, that the nervous centres may be aptly compared to a galvanic apparatus, Dr. *Baillarger* next alludes to the minute anatomy of the medullary matter; and suggests that the innumerable fibres, which it everywhere sends into the cineritious matter, may be regarded as so many conducting points which draw off the nervous or galvanic power.

The following paragraph is interesting.—

"If we consider that in those animals, which are highest in the scale of intelligence, the brain is the most convoluted, and presents therefore the largest extent of surface ; and if to this consideration we add that not only is delirium much more frequent in diseases of the superficial than of the deep-seated parts of this organ, but also that in dementia it is usually the cortical substance that is found atrophied or otherwise diseased, we cannot hesitate to attribute important functions to the cerebral *surfaces:* indeed they seem to be the parts that are most essential to the performance of the functions of the nervous system."

Appended to this memoir are two lithographed engravings, which beautifully illustrate the stratified structure—consisting of six layers alternately of white and cineritious matter—of the cortical substance of the cerebral convolutions. The memoir is altogether well worthy of the attention of the physiologist, and will doubtless attract the notice of subsequent writers on the anatomy of the nervous system.

The next memoir is a most lengthy and elaborate one on the

*Statistics of Acute Pleuropneumonia* by Dr. *Pelletan*, chief clinical clerk of M. *Bouillaud* at the Hôpital de la Charité. As we have noticed its leading conclusions in the Foreign Periscope of the present number, we have no intention of enlarging upon the subject at present. Besides, the article itself is one of those most wearisomely and unnecessarily minute productions which issue so frequently from the *Bouillaud* section of physicians in France. Thus seventy-five cases, the reports of which occupy nearly fifty pages, occupy the first part, and other forty pages are taken up with tabular views and comparisons. Our readers are aware that we are no admirers of the arithmetical school of physicians, who strive so hard to reduce the phenomena of diseases and the principles of their treatment to certain rules, which may have all the exactitude of numerical calculation. Medicine, no doubt, may be improved and simplified, and much, it will be admitted, has been done during the present century to effect this important end; but no rational man, we think, can expect that ever it will be reduced to the certainty of an exact science. The very nature of an organized living frame, as that of man, made up of such varied and such complicated machinery, and obnoxious to so mysterious and never-ceasing a variety of internal and external agencies, all tending to disarrange its wonderful economy, must for ever forbid this Utopian fancy. Let it however not be imagined for a moment that we are at all inclined to disparage the usefulness of statistical tables of diseases, or to condemn any attempts to discover the comparative value of different lines of treatment by such arithmetical calculations as M. *Bouillaud* and his admirers so enthusiastically recommend. But we have seen quite enough of medical practice to be assured that no two cases of the same disease are in every respect and entirely alike, or that exactly the same line of treatment is equally adapted to each. Every case of disease has its peculiarities, and requires its special modifications of treatment. To expect therefore that we shall ever be able to lay down precise rules as to the amount of blood to be drawn, or, to use the French expression, *formuler la saignée*, in a disease like pneumonia, and these rules to be applicable to all patients, and in all seasons and years alike, is at once chimerical and dangerous.

But we must now pass on to notice the series of—

*Memoirs on Poisoning*, by the distinguished toxicologist of France, M. *Orfila*. Our limited space utterly precludes us from following him through these elaborate and most valuable papers; and even if we had more room at our command, it would be impossible to do justice to them within the ordinary bounds of a single article. Suffice it therefore to say that, in the first place, he has described a vast number of experiments on dogs, and also a few which he has made on the bodies of human beings who have died from the effects of arsenical poisoning: for the details of these we must refer the reader to the original. The following are some of the important conclusions which he has deduced from his laborious researches.*

* We have already briefly noticed them in this Review for last January—vide Foreign Periscope, p. 226.

1. That arsenious acid, introduced into the stomach or applied to the subcutaneous cellular substance in dogs, is quickly absorbed and received into the circulating fluids; and that it is thus conveyed to, and penetrates, every part of the animal economy.

2. That when it is applied in the state of a fine powder to the cellular tissue, there is not more than from one grain and a half to two grains actually absorbed, however large the quantity that may have been applied; and that this small quantity is sufficient to cause death, since it is impossible to attribute this result to the local irritation, which is usually inconsiderable.

3. That more is absorbed, although the precise quantity cannot be ascertained, when the poison is introduced into the stomach.

4. That arsenious acid acts in a similar manner in the case of human beings, although we may presume that a larger portion requires to be absorbed to occasion death with them than with dogs.

5. That, it is practicable, by the aid of certain chemical processes, to detect the arsenic which has been thus absorbed.

6. That, for this purpose, experiments must be made separately on the blood, the viscera, and the urine; since, at a certain period after the poison has been absorbed, it is no longer discoverable in the blood, although it still exists in notable quantity in the substance of the viscera, and in the urine; and at a later period it can no longer be detected in the viscera, while it may still be found in the urine. On two occasions, by examining the blood drawn from patients fourteen hours after the swallowing of the poison, a sufficiently large quantity of metallic arsenic was obtained from it to satisfy us that probably traces of its presence might have been appreciable, even if the analysis had not taken place for ten or fifteen hours later; and as to the viscera, it has been most distinctly proved that the arsenic may be discovered in them for several days afterwards.

7. That if, against all probability, no arsenic can be detected in the blood, viscera, or even in the urine which may be in the bladder at the moment of death, we are not to conclude with certainty that there has not been any poisoning at all; for it is quite possible that the absorbed poison may have been completely eliminated and discharged with the urine passed during the life of the patient: hence the necessity of preserving all the urine which may be voided by any person who is suspected of having taken arsenic.

8. That to extract from the blood a sufficient quantity of arsenic, we do not require more than a few ounces; and therefore that it will be judicious, independently of the propriety of the practice to subdue any existing inflammatory symptoms, to bleed every suspected patient, for the purpose of obtaining blood for chemical analysis.

9. That, although we may detect in the majority of cases the arsenious acid in one of the very vascular viscera, such as the spleen or liver, which has been previously dried, it is always preferable to act upon several of them at the same time, as the proportion of the absorbed poison may be too minute to warrant a decided affirmation of its presence in any one.

The process recommended by *Orfila* to detect the presence of arsenious acid in the blood and in the substance of the viscera, is to make a strong decoction of them, previously dried and cut into small pieces, in pure distilled water, to treat this with sulfhydric acid, evaporate it to dryness, then carbonise the residue with pure concentrated nitric acid, submit the carbonised mass to the action of boiling water, and introduce the filtered fluid into Marsh's apparatus.

In this way, by treating *half only* of the liver of M. *Lorrin* who poisoned himself in June of last year, he discovered distinct traces of the existence of arsenic; and the same satisfactory results were obtained in a still more remarkable case, that of *Nicolas Mercier* who had been buried upwards of five months before the body was examined. By carbonising the liver with nitric acid, and boiling the charred mass in distilled water, and then introducing the filtered liquor into Marsh's apparatus, the presence of arsenic was ascertained beyond the possibility of doubt.* In the following case, in which the patient recovered after swallowing nearly a coffee-spoonful of arsenious acid, the presence of the metal was detected in the blood which had been drawn from the arm for the purpose of relieving the inflammatory symptoms. It was first dried in a porcelain vessel with four grains of pure potassa; the dried mass, which weighed about two ounces and a half, was then decomposed by means of five ounces of the purest nitric acid; it became immediately charred. The carbonised mass, bulky and dry, was boiled in distilled water, and when this was introduced into Marsh's apparatus, at least a score of minute but brilliant arsenical spots appeared. Besides the cases now mentioned, others have occurred since the publication of M. Orfila's memoir, in which the detection of arsenic in the bodies of persons who had died, or were suspected to have died, from the administration of this poison, was most satisfactorily made out by the process which we have described: in one instance the detection took place fifteen months after the decease of the person. Well, therefore, may M. *Orfila* say—

"In future, crime will often be pursued with success even into its deepest and last refuge; for without doubt several of the poisons, which act by absorption, will henceforth be detected in the tissues of the animal economy. Researches undertaken with this view and founded on the experiments which I have recorded will ere long resolve this great problem of legal medicine, and will doubtless at the same time explain and illustrate many obscure points in physiology and therapeutics."

M. *Orfila* proceeds to detail a series of experiments performed on dogs by which he has been able to prove most distinctly—1. That in poisoning with the tartrate of Antimony, whether it be introduced into the stomach or applied to a wound, this salt also is quickly absorbed into the blood and penetrates the tissue of the viscera, where it remains only a short time, especially if these viscera are not secreting organs; and—2. That, after having left the viscera, it is eliminated with the urine and probably also with some of the other secretions. The rapidity, with which the antimonial salt is absorbed into and eliminated from the system, is much greater than in the case of the arsenious acid: thus after the lapse of even a few hours no traces of it can be detected in the blood, while it may still be distinctly discovered in the viscera, especially the liver and kidneys, and in the urine.

---

* The reader is referred to another case, detailed in the Foreign Periscope of this Review for January of last year, where the presence of arsenic was detected in a corpse three years after interment.

The process recommended by M. *Orfila* is very nearly the same as that which we have described for the detection of arsenic: the dried viscus or viscera are charred with pure nitric acid, and the carbonised mass is boiled for half an hour with hydrochloric acid; the solution, filtered, is then introduced into Marsh's apparatus: antimoniated hydrogen is quickly evolved, and if this be inflamed a great portion of the metal is deposited. For a full account of the discriminating characters between the spots or traces left by the two metals, antimony and arsenic, we must refer our readers to the original memoirs.

In several cases M. *Orfila* has succeeded in detecting the presence of antimony in the urine of the human subject. 1. In the case of a patient affected with pneumonia and to whom 120 centigrammes* of the tartrate of antimony had been administered in the course of 24 hours, he was able to obtain a sufficient quantity of the metal from the urine, to exhibit it to the members of the Academy in their sitting last April. 2. In another case, which occurred in an old woman who had taken 60 centigrammes of the salt in the course of 24 hours without its producing either vomiting or purging, the urine, voided twelve hours after the administration, being evaporated, carbonised, and then treated in the manner already described, afforded unequivocal traces of the presence of the metal. 3. The liver, kidneys, and spleen taken from an aged inmate of the Salpetriere, who died fifteen hours after having taken five decigrammes of the tartrate, yielded distinct traces of antimony.

M. *Orfila* closes his remarks in the following words:

"If the recent experiments of M. Blacke prove that certain very active vegetable poisons are absorbed and penetrate all the organs of the body in the course of a few seconds—*Majendie* had already observed this with phosphorus—it follows from mine, that the antimonial tartrate after its absorption does not remain in the blood, or at least not in a quantity sufficiently appreciable to be detected by Marsh's apparatus, more than one hour after the poisoning has taken place.

The arsenious acid remains very considerably longer in the circulating fluids; as I have been able to detect it in the blood of dogs three hours afterwards, and in the urine of the human subject at least 13 hours after the administration of the poison. But however this may be, it is curious, although not surprising, to find that the antimonial and arsenical salts, after having left the blood and being deposited in different tissues of the body, remain much longer and in larger quantities in the secretory than in any of the other viscera, before they are completely eliminated from these viscera into the secreted fluids."

In a subsequent memoir our indefatigable author pursues a similar line of enquiry with respect to the salts of Copper, as he had already done with those of Arsenic and Antimony; and he most satisfactorily proves that this metal also may be detected in the fluids, the viscera, and some of the secretions, several hours after one of its salts, as the sulphate or the acetate, has been taken into the stomach. We regret that we cannot afford space at present for further details, and must therefore urgently recommend all who take an interest in chemistry, physiology, or in legal medicine, to peruse and study

---

* For an account of the decimal system of weights now used in France, vide the Foreign Periscope of the present number.

the original memoirs for themselves. To the practical physician they suggest many topics for instructive reflection; more particularly as shewing him with what rapidity certain metallic remedies, which he is in the daily habit of prescribing in various diseases, are absorbed into the circulation and conveyed into the texture of the different organs of the body. The science of therapeutics cannot fail to receive many curious and useful hints from such a consideration.

The remaining memoirs in this volume will not detain us long. There is a long, and we had hoped an interesting, one on the important subject of re-vaccination by M. *Sedillot,* the President of the Vaccine Commmittee. It extends to upwards of 100 pages, and yet we may safely assert that it does not communicate a single novel fact nor indeed any series of valuable data on the subject which it professes to treat of. The greater part of it is occupied with a most unnecessary description of variola, varicella and vaccinia. The author denies that small-pox ever occurs in the same individual twice,* or even that it ever occurs in a person who has passed through the *genuine* cow-pox. He asserts that the preservative power of the latter against the former disease is *absolue et illimitée ;* and he therefore condemns all proposals to re-vaccinate as not only quite unnecessary, but as hurtful by disseminating the idea that the protection afforded to the vaccinated person is only temporary. We have no intention to canvas the various *assertions,* for they are nothing else, of M. *Sedillot,* and will refer our readers to a carefully drawn up article on the subject of vaccination and re-vaccination in the number of this Journal for April of last year.†

The value of M. *Lecanu's* paper, entitled,

*New Researches on Human Urine,* may be judged of from the following conclusions with which he sums it up:—

1. The urea is secreted in equal quantities, during equal periods of time, by the same individual.

2. The uric acid also is secreted in equal quantities, during equal periods of time, by the same individual.

3. The urea and the uric acid are secreted in different quantities, during equal periods of time, by different individuals.

4. The variable quantities of urea, secreted by different individuals during equal periods of time, are proportionate to the age and sex of the persons —being greater in men than in women, and in women than in old persons and children.

---

* M. *Sedillot* mentions, as a most convincing proof of the accuracy of this statement, that the sum of 12,000 francs were deposited in the hands of the receiver-general of finances at Paris by a M. *Gatti,* to be given to any one who should produce a case of the genuine recurrence of small-pox, and that the reward has not been claimed by any one. (It is not said whether the offer has been withdrawn.) We have however the authority of Dr. *Gregory,* and indeed of almost every author who has written on small-pox, that second attacks of small-pox in the same individual do occur occasionally: vide also the *Medico-Chirurgical Review,* for last July, p. 516.

† In the numbers also for July, p. 193, and for October, p. 555, 1839, will be found some instructive extracts, from the writings of several authors both in France and Germany, upon this most interesting subject.

5. Those constituents of the urine which are fixed and undecomposable by heat, such as the earthy phosphates, the chloride of sodium, and the alkaline sulphates and phosphates, are secreted in different quantities, not only in different individuals, without having any relation to age or sex, but also in the same individual during equal periods of time.

The accuracy of these conclusions being established, " we are enabled to determine," says M. *Lecanu*, " 1, if the secretion of the urea and uric acid in any patient continues, or not, to go on regularly as in a state of health; 2, if in the same patient the secretion of these elements continues, or not, within the ordinary limits which it exhibits in healthy individuals of the same age and sex; and lastly, if under the influence of disease and the treatment which is adopted for its relief, the secretion of the urea, the uric acid, and of the saline matters experiences, or not, any changes—during equal periods of time, as for example during 24 or 48 hours—in reference either to the manner in which it is effected, or to the quantity of each of the elements which it produces."

The last Memoir is entitled—

*Vaginal Cystocele operated on in a New Method*, and is communicated by M. *Jobert*, one of the surgeons of the St. Louis Hospital in Paris. The following extract will enable our readers to judge of the operation.

......" I applied myself to effect a diminution of the tumor without causing almost any loss of substance in the part, and without exposing the patient to the risk of much hæmorrhage. These results I hoped to obtain in the following manner. With the nitrate of silver I marked out upon the tumor two transverse lines about half an inch wide, and about an inch apart from each other, and by repeating the application several times, at intervals of a few days, I at length destroyed, gradually and without any inflammatory accident, the entire thickness of the wall of the vagina in the cauterised portions. This being effected, I then with a bistoury cut (ravivais) the edges of the surfaces which had been cauterised. The intermediate space between the two incisions being pushed upwards, the bleeding surfaces were easily brought together and retained thus by means of a twisted suture. The seven needles which I used were contained in sheaths or canulæ, so that they (the needles) might be withdrawn, and the sheaths only be left in their place for the application of the ligatures."

To prevent the contraction of the bladder during the process of healing, an elastic gum catheter was introduced and left in it. The sheaths were withdrawn, some on the seventh, and the others on the tenth and twelfth days after the operation. The result of the case was most satisfactory, the woman being able to walk, and ultimately to resume her employment as a washerwoman, without any return of the tumor.

M. *Jobert* has repeated the operation in a second case with equal success. He has also succeeded by the same method in effecting a cure in several cases of prolapsus of the uterus accompanied with great relaxation of the parietes of the vagina.

